# Students’, parents’ and teachers’ perspectives on comprehensive school-based sleep promotion

**DOI:** 10.1177/00178969241286660

**Published:** 2024-09-30

**Authors:** Pamela Mellon, Genevieve Montemurro, Samuel (Sukmin) Yang, Lauren Sulz, Brian Torrance, Kate Storey

**Affiliations:** a School of Public Health, University of Alberta, Edmonton, AB, Canada; b Faculty of Medicine and Dentistry, University of Alberta, Edmonton, AB, Canada; c Faculty of Education, University of Alberta, Edmonton, AB, Canada; d Ever Active Schools, Edmonton, AB, Canada

**Keywords:** Community, home–school collaboration, qualitative research, school culture, sleep promotion

## Abstract

**Objective:**

Sleep deprivation is common among children and schools are ideal settings in which to influence children’s sleep. Children spend a significant amount of time at school during key developmental periods, and programmes that influence students’ well-being also benefit academic achievement. Comprehensive School Health (CSH) is an approach that prioritises school, home and community partnerships by supporting the development of health behaviours. However, sleep is often unaddressed in the school environment. The objective of this study was to integrate multiple partner perspectives to inform how to strengthen school-based sleep promotion using a CSH approach.

**Design:**

A secondary qualitative analysis of student aged 9–11 (*n* = 45), parent/guardian (*n* = 24) and teacher (*n* = 19) interviews from participants representing elementary (Kindergarten – Grade 6) schools and communities in Alberta, Canada was conducted. Data were examined using an *a priori* framework in alignment with the four components of the CSH approach: social and physical environment, teaching and learning, policy and partnerships and services. Inductive content analysis was used to develop categories followed by subthemes within each CSH component.

**Results:**

Subthemes identified within each component were as follows: social and physical environment (culture of healthy sleep habits; students influence each other); teaching and learning (formally integrate sleep-specific learning into curricula; school, teacher and parents/guardian collaboration); policy (sleep-positive classroom policies) and partnerships and services (community partnerships; school–home collaboration).

**Conclusion:**

This research identifies recommendations from student, parent/guardian and teacher interviews to inform and strengthen school-based sleep promotion when taking a CSH approach. Findings can support school partner efforts to foster a school culture (or ethos) of healthy sleep habits leading to improvements in student sleep behaviours.

## Introduction

Sleep is a biological imperative that is required for health (Cirelli and Tononi, 2008), yet we live in a society that is not well aligned with this biological drive (Grandner, 2017). Inadequate sleep is so pervasive in our society that it is regularly overlooked as the potential cause, barrier or comorbid factor of presenting health concerns (Chaput et al., 2022). Rates of insomnia in Canada have doubled in the past 20 years (Chaput et al., 2022), with 50% of adult residents regularly struggling to fall or stay asleep (Chaput et al., 2017). Children and adolescents are not exempt from this ‘sleepidemic’ (ParticipACTION, 2016), as approximately 30% of children and youth in Canada do not meet sleep recommendations according to the 24-hour Movement Guidelines for children and youth, which recommends that children (5–13 year) and youth (14–17 years) sleep 9–11 hours and 8–10 hours per night, respectively (ParticipACTION, 2022). Inadequate sleep affects learning, school performance, emotional regulation and physical health (Matricciani et al., 2019). Inadequate sleep among childhood has the potential to jeopardise children and youth’s academic, vocational and physical potential (Chaput et al., 2022).

Adequate sleep is essential for learning and academic success in children, and schools can play a significant role in promoting healthy sleep behaviours among students (Chaput et al., 2022). Since the origins of the World Health Organisation’s Global School Health Initiative in 1995 (O’Byrne et al., 1996), many jurisdictions internationally have embraced Comprehensive School Health (CSH) or its equivalents (e.g. Health Promoting Schools or Whole School, Whole Community, Whole Child) as recognised approaches to promoting health behaviours in schools (JCSH, 2006, 2019; Langford et al., 2014). Whole-school models to address school health build capacity in school communities (school, home and community) to promote health and well-being alongside educational attainment. In Canada, existing CSH efforts have proved effective at improving children’s healthy eating and physical activity behaviours (Dabravolskaj et al., 2020); however, sleep promotion is behind (Chaput et al., 2022).

CSH addresses health behaviours in four ways: through social and physical environments, teaching and learning, policy and partnerships and services (JCSH, 2016). Social environments refer to relationships in schools among and between staff and students as well as the emotional well-being of students (Ever Active Schools, n.d.). Physical environments refer to the buildings, grounds, play spaces and equipment both in and surrounding the school (Ever Active Schools, n.d.). Teaching and learning includes the ways that students learn and practise health at school including curriculum-based instruction and activities, informal instruction and professional development for school staff (Alberta Health Services, n.d.). Policy refers to ‘policies, guidelines and practices that promote and support student well-being and achievement, and shape a respectful, welcoming and caring school environment for all members of the school community (Alberta Health Services, n.d.)’. Partnerships are connections and relationships between schools and communities (i.e. parents/guardians, families, community organisations, elders and local recreation facilities), whereas services are described as the supports offered by health professionals or social services providers (i.e. immunisations, teacher training, presentations, resources and coaching) (Alberta Health Services, n.d.). In addition to the four components, CSH provides the opportunity to address inadequate sleep among children and youth through a whole school approach which includes a focus on the school, home and community environments (JCSH, 2016).

Existing school-based sleep intervention efforts prioritise sleep education, which include in-class educational components and behavioural change strategies (Ashton, 2017; Davidson et al., 2022; Gruber et al., 2016; Rey et al., 2020). School-based sleep education programmes have been found to regularly increase students sleep knowledge; however, they are largely ineffective in improving students’ sleep behaviours (Gruber, 2017, 2020; Rigney et al., 2021). Typically, school-based sleep education programmes are facilitated by external partners and have been unsustainable without continued presence of the research team (Gruber et al., 2017). As such, researchers working to promote school-based sleep efforts advocate for interventions to be facilitated by teachers (Rey et al., 2020), integrated into existing health education curricula time (Chaput et al., 2022) and to take a comprehensive and collaborative approach, involving school community partners (i.e. families, teachers, administrators and community agencies) (Gruber et al., 2017).

Bird et al. (2021) found that as sleep happens in the home environment, parent involvement is necessary for school-based sleep promotion to be successful. In addition, Bird et al. (2023) found that parents are willing to be involved in school-based sleep promotion and see school-based sleep promotion as a strategy to reach families that do not promote healthy sleep behaviours at home. Finally, Mellon et al. (2024) found that teachers (teachers and administrators) are supportive of home–school collaboration to promote healthy sleep behaviours for students. For the purposes of this paper, the term ‘school-based sleep promotion’ refers to strategies that support positive sleep behaviours for children and families that originate in schools.

Understanding student, parent and teacher perspectives regarding school-based sleep promotion would provide a nuanced understanding of the school’s role in promoting healthy sleep behaviours in students. Understanding student, parent and teacher perspectives on school-based sleep promotion through a CSH approach would allow school-based sleep promotion to be understood through a common framework. CSH is already recognised across Canada and internationally; however, there is less known about how the CSH approach can be used to promote healthy sleep behaviours. Aligning school-based sleep promotion with the CSH approach would promote a shared understanding, practical strategies and insight into implementing school-based sleep promotion in school communities across Canada and beyond.

To address this gap, the objective of this research was to integrate multiple partner perspectives to inform how school-based sleep promotion can be strengthened when taking a CSH approach. Using a secondary analysis of qualitative interview data, we sought to understand how different school partners (students, parents/guardians and teachers) perceived school-based sleep promotion with specific attention to alignment with the four components of CSH. Data from three previously published studies with different participant groups (Bird et al., 2021, 2023; Mellon et al., 2024) were analysed with a goal to inform and strengthen school-based sleep promotion through the identification of existing beliefs, behaviours, practises, strategies or recommendations to promote healthy sleep behaviours in students.

## Methods

### Data collection

Qualitative secondary analysis can be used to investigate new or additional research questions from pre-existing data sets. This qualitative secondary analysis used previously collected interview data from a larger research project that examined (1) how students aged 9–11 translate school-based sleep promotion to the home environment; (2) parents’ perspectives on sleep behaviours and their role in promoting healthy sleep behaviours; (3) teachers’ perspectives on sleep behaviours and their role in school-based sleep promotion, and; (4) school-based sleep promotion when taking a CSH approach.

Three separate studies examining different partners’ (i.e. students, parent/guardians and teachers) perspectives on school-based sleep promotion were combined and examined for this study. Each of the three studies was driven by community-based participatory research, which has its origins in ethnography and interpretive analysis (Mayan, 2016), and they are therefore aligned methodologically. While the purpose of each study differed, the overarching objective of all the studies was to understand how to improve student sleep behaviours. As data sets were methodologically aligned in this objective, participants were purposively sampled, and the researchers were responsive and reflexive by revisiting themes, peer debriefing, and staying true to the iterative nature of the research.

### Primary datasets: student, parent/guardian and teacher interviews

The larger research project was composed of three separate studies, of which the datasets were used for this research.

The purpose of the first study was to explore ‘student’s perceptions of sleep behaviour (how they understood and valued positive and negative sleep behaviours) and determined if and how students translate school-based sleep promotion to the home’ (Bird et al., 2021: 588). Forty-five grade 4 and 5 (ages 9–11 years) students from three schools in Edmonton, Canada participated. The guiding method was focused ethnography, with photovoice as the data generating strategy. In line with photovoice guidelines, semistructured interviews were conducted with all 45 young people. The purpose of the second study was to ‘explore parents’ perspectives on sleep behaviours and responsiveness to school-based sleep promotion’ (Bird et al., 2023: 1). Twenty-four parents/guardians of school-aged children (ages 5–12 years) from across Alberta participated in a descriptive qualitative study in which semistructured interviews were used as the data generating strategy. The third study was conducted with 19 elementary teachers from the greater Edmonton area and interpretive description was the method used to ‘explore teachers’ perspectives on sleep behaviours and their role in school-based sleep promotion’ (Mellon et al., 2024: 357).

All three studies aimed to understand partners’ perspectives of students’ sleep behaviours and school-based sleep promotion initiatives. All interviews were audio-recorded and transcribed verbatim by a professional transcriptionist, and all studies were granted ethical approval by the Human Research Ethics Board at the University of Alberta (Reference: Pro00078831) which included approval for secondary analysis. K.S. was the academic supervisor on all three studies, while two graduate trainees were the respective lead authors. The lead author of this secondary analysis (P.M.) supported data generation for parent interviews and conducted teacher interviews. Participants were allocated a code to represent student, parent/guardian or teacher interviews (e.g. S1, P1, T1), all other identifying information was removed.

### Secondary data analysis

All 88 interview transcripts were read and re-read by the first and second authors. Data were then analysed using content analysis comprised of three steps: (1) data reduction, (2) data display and (3) conclusion drawing/verification (Miles et al., 2014). First using a deductive approach, transcripts were broadly coded into an *a priori* framework (matrix) by P.M. and S.Y. according to the four components of CSH (social and physical environment, teaching and learning, policy and partnerships and services), while maintaining participant groups. This matrix informed initial interpretations and prompted important early analytic debriefing. Early development and refinement of the matrix was supported by a reference table describing the key characteristics and published definitions for each of the four CSH components (Alberta Health Services, n.d.; Ever Active Schools, n.d.; JCSH, 2016). This reference table is provided as an online supplemental table (Supplemental Table 1). As CSH components often overlap in school environments, distinct definitions of the components were necessary to support further deductive analysis. Using a deductive approach, data were further condensed (coded) according to the four CSH components and organised using NVivo 12 (QSR International Pty Ltd., 2018).

Next, inductive content analysis was used to develop categories within the CSH components and participant groups (Miles et al., 2014). Inductive content analysis allowed new concepts to be developed from the student, parent/guardian, and teacher perspectives (Kyngäs, 2020). Categories were then merged across participant groups, leaving data grouped by CSH components. Inductive content analysis including data display (i.e. lists, infographics) was further used to develop subthemes within each CSH component. Peer debriefing was used throughout analysis to support verification of categories, subthemes and themes. The initial deductive approach provided a valuable method of organising the data themes around the CSH components, while the inductive approach allowed for new insights to be identified from students, parents/guardians, and teachers. Due to the nature of organising secondary data into an a priori framework (CSH), varying levels of student, parent and teacher perspectives were identified within the secondary data sets in relation to school-based sleep promotion and the CSH components. Our analytic approach focused on the overall integration of partner perspectives across themes and subthemes, with attention to which partner perspectives were represented across components.

## Results

This study integrated student, parent/guardian, and teacher perspectives to inform and strengthen school-based sleep promotion when taking a CSH approach. The following subthemes were identified within the four CSH components: (1) social and physical environment (culture of healthy sleep habits; students influence each other); (2) teaching and learning (formally integrate sleep**-**specific learning into curricula; school, teacher, and parents/guardian collaboration); (3) policy (sleep-positive classroom policies), and: (4) partnerships and services (community partnerships; school–home collaboration).

### Social and physical environment

The social and physical environment of a school plays an important role in establishing a healthy school culture (or ethos), as culture is influenced by both the social (i.e. conversations, relationships, experiences, teaching and learning) and physical (i.e. facilities, classrooms, gymnasium and playground) environments. Teachers shared the ways in which they saw the school community build a culture of healthy sleep habits. Teachers, students and parents/guardians illustrated how students are change makers regarding healthy sleep behaviours in both the home and school environments. This theme was supported by two subthemes: creating a culture of healthy sleep habits and students influence each other.

#### Creating a culture of healthy sleep habits

Teachers recognised themselves as role models and acknowledged that how they talked about sleep influenced students’ perspectives on sleep. For instance, teachers shared how they celebrated with the class when they had a good night’s sleep and shared when they had slept poorly. Some teachers brainstormed with students about how to get better sleep. Through conversations with students, teachers demonstrated that they valued sleep and felt their best when they got enough sleep:

I’ll tell them I’m kind of feeling tired cause I went to bed a little late or I couldn’t sleep because this happened. In a lot of health classes, we start with just a discussion, whether it’s about nutrition or sleep or fitness. And then they’ll kind of share what they do. (T11)

Teachers described many occasions where they had informal conversations with students about sleep (i.e. late to school, falling asleep in class). Connecting with students first (before parents) about their sleep habits was recommended by teachers, as older students were more likely to change their sleep behaviours if they were involved in the decision-making process. Class discussions and informal conversations were observed by teachers to influence the social environment of the school, including students’ perspectives on healthy sleep behaviours.

Monthly campaigns (i.e. bulletin boards, announcements and lessons) were perceived by teachers as a way for the whole school to have a universal language to talk about a concept like sleep. One teacher shared how monthly campaigns helped the topic ‘ripple across the whole school’ (T13). Teachers shared that when administrators promoted sleep-related school-wide initiatives, they were perceived as sending a message that sleep is important, ‘if it’s promoted schoolwide, it’s continued. If you’re showing as an administrator that it’s important, if I’m showing it’s important to me, and I believe it’s important to kids, then I feel that belief is filtered down’ (T7). Bulletin boards were viewed as good places to highlight fun sleep facts and student sleep habits. One teacher explained how older students in grades five and six shared how much sleep they get and their bedtime on the bulletin board, which inspired younger students to get more sleep. Bulletin boards and announcements also influenced conversations that students had with each other.

#### Students influence each other

Teachers observed how students played an important role in school-based sleep promotion, as older students taught younger students about healthy sleep habits through buddy classes, that is, classes partnering up for a lesson, s)/activity) or bulletin boards. Students learned from each other about sleep habits through class discussion or informal conversations, and some students took what they learned at school back to their home environment, to create healthy sleep habits:

My sister she doesn’t really want to go to bed when she’s supposed to. I sometimes say it helps you sleep better. I tell her that she has to go to bed so she won’t be grumpy in the morning and so she’ll have a good day. (S32)

Teachers emphasised that students were able to ‘bring ideas to life’ and drove initiatives forward. A few students, parents/guardians and teachers explained how some students incorporated what they learned about sleep at school into their sleep habits. One student shared how ‘I try to encourage myself to sleep better because I remember the lessons that we’ve had at school’ (S51). Students and teachers expressed how students influence each other, both positively and negatively, through interactions related to sleep. For instance, some students shared unhealthy sleep habits like bringing screens into bed or staying up till 2:00 a.m. gaming. In contrast, other students shared their bedtime and encouraged each other to get to bed early so they could have fun together the next day. Students and teachers described sleep-related monthly campaigns and teachers emphasised how students ‘can’t help but really just talk about what’s going on in their environment every day’ (T5). Teachers highlighted how students were more likely to try a sleep tip from a peer than an adult. As one teacher shared, ‘. . .with their friends, they think, okay, if that person’s doing it, I could try it’ (T11).

### Teaching and learning

Teaching and learning using a CSH approach directs attention towards formal and informal curricula, resources, and associated activities. Rather than sleep being part of the informal curriculum as it is now, parents/guardians and teachers felt that sleep should be formally integrated into the curriculum. Teachers and parents/guardians also supported the idea of collaboration between schools, teachers and parents/guardians to support healthy sleep behaviours in students. According to students, they learned about sleep from home and school. As such, this theme comprised two subthemes: formally integrate sleep-specific learning into curricula and school, teacher and parent/guardian collaboration.

#### Formally integrate sleep-specific learning into curricula

Teachers and parents/guardians viewed sleep as important and thought that a focus on sleep was already present in the provincial school curricula or that sleep should be part of the provincial curriculum: ‘. . .[sleep] should be in the curriculum, in the health curriculum too because sleep is important; even if it’s just like a day of teaching about sleep’. (P48). One teacher stressed how ‘I’m sure [sleep’s] part of the health curriculum, or you could find a connection to the health curriculum. But if [teachers] don’t feel it’s important, they’re not going to make time for it’ (T7). Currently in schools, according to teachers, sleep is covered if the school or teacher sees sleep as a priority and chooses to make a relevant curricular connection.

#### School, teacher and parent/guardian collaboration

Teachers and parents/guardians advocated for collaboration between schools, teachers and parents/guardians to promote healthy sleep habits. While sleep happens at home, students spend many hours in the day at school learning and having conversations with fellow students, teachers, administrators and other school staff. According to parents/guardians, collaboration between school and home allowed students to hear the same message from multiple adults, which reinforced the message that sleep is important.

One parent/guardian explained how ‘[students are] going to have a bigger buy-in hearing it from mom, dad, and teachers. Especially if it’s told at school. It must be true . . . So yeah, I think parents need that kind of support’ (P16). Parents/guardians were supportive of teachers educating about the importance of sleep and healthy sleep habits as teachers have influence on their children. One parent/guardian described an anticipated benefit if schools educated students about healthy sleep behaviours:

I think that would help the kids understand why we’re making them go to bed at certain times or why we are shutting electronics off . . . I know that a lot of kids don’t get that from their parents, so I know that if the school were to help out a little bit teaching that, I think that it would be very good. (P49)

Teachers agreed that they had a role to play in promoting healthy sleep behaviours in schools. Teachers recognised that doing so must however be a team effort between students, parents/guardians and teachers to improve students’ sleep behaviours:

I think that teachers absolutely do have a role. I don’t know if I would say that we have the biggest role. I think a lot of that responsibility does have to stay with the family, but I do absolutely think that teachers have a role to be educating the students and then the students have a role in taking responsibility for themselves to a certain degree. (T3)

### Policy

No formal school or district-level policies were described by participants regarding sleep and sleep education. Classroom policy related to sleep was touched on by the teachers interviewed, specifically as it related to letting students sleep when tired.

#### Sleep-positive classroom policies

Across participants (students, parents/guardians and teachers), there was a clear understanding that tired students had increased difficulty learning, staying engaged and interacting with their peers at school. Teachers shared how they let students sleep whenever possible, as often students ‘need rest more than anything’ (T13). Teachers acknowledged that students often fell asleep at their desks, slept in the sick room or fell asleep after being sent to the principal’s office due to being emotionally overwhelmed. Teachers perceived it to be best if students got enough sleep at home; however, this was not possible for all students. One teacher shared how allowing students to have a nap rather than sending them home meant that students had more opportunities to learn. Teachers recognised that students often woke up refreshed, better ready to learn and socialise at school. As one teacher described, ‘if they’re still sleeping and there’s all this noise going on, they must need it’ (T14). School practises shared by teachers to support healthy sleep behaviours in students included monthly campaigns on sleep and connecting with parents/guardians when students are dysregulated, falling asleep in class or falling behind in schoolwork. Teachers encouraged connecting with parents/guardians if initial attempts with the student did not correct sleep behaviours or when students being emotionally dysregulated, sleepy or falling asleep in class was a recurring problem.

### Partnerships and services

Partnerships and the services available to support individual school communities depend on community context. Partnerships are connections and relationships between schools and communities (i.e. parents/guardians, families, community organisations, Elders and local recreation facilities), while services may be described as supports offered by health professionals or social service providers (i.e. immunisations, teacher training, presentations, resources and coaching) (Alberta Health Services, n.d.). According to parents/guardians and teachers, community partnerships that share resources and knowledge about healthy sleep behaviours are limited despite teacher and parent/guardian interest. Meanwhile, school–home collaboration is a critical partnership for healthy sleep according to parents/guardians and teachers, as schools and home influence children’s sleep. This theme was supported by two subthemes: community partnerships and school–home collaboration.

#### Community partnerships

Teachers and parents/guardians observed that schools can be a hub for partnerships. However, sleep-related community partnerships and services to strengthen school-based sleep promotion were limited. Community partnerships mentioned by teachers that supported teaching students and families healthy sleep behaviours included the APPLE Schools programme (APPLE Schools, 2022), family school liaison workers or community outreach staff in the school. Programmes such as Leader in Me (Franklin Covey Company, 2022) and Zones of Regulation (Kuypers, 2011) were mentioned by teachers as providing opportunities to support promoting healthy sleep behaviours. Several organisations had resources that teachers accessed through the Internet (e.g. Alberta Health Services, Alberta Health, ParticipACTION, Canadian Sleep Society). Students also shared how after school sports or clubs helped to tyre them out for bedtime. Parents/guardians and teachers encouraged further engagement with partnerships and services to support school-based sleep promotion.

#### School–home collaboration

Teachers described existing school-home collaboration for healthy sleep habits as including parent–teacher interviews, newsletters, school website, parent council events, phone calls and emails. However, parents/guardians and teachers encouraged more collaboration between school and home to teach students healthy sleep habits. For instance, some teachers wanted to know when students were having sleep problems so they could have more patience when the student was falling behind or misbehaving. Parents/guardians perceived teachers to be a good resource to help students understand that sleep is important. Parents/guardians and teachers shared that parent/guardian engagement in school-based sleep promotion could include schools sharing resources and community contacts with families to support students healthy sleep behaviours. Parents/guardians encouraged schools to share resources and guide parents/guardians towards resources on children’s healthy sleep behaviours, ‘cause I don’t think parents are going to go do it on their own’ (P52). Other suggestions from teachers included a school-wide sleep awareness event (i.e. family night, open house and resource fair). Both teachers and parents/guardians highlighted a need for parent/guardian education as many parents/guardians were unaware of how much sleep their children need:

I really feel like there needs to be parent education as well as student education because something that I’ve noticed is it seems around this age, when they’re in grade three and four, parents’ kind of hand over the reins a little bit more to the students. Which is great to be giving them more responsibility and things, but I also think they’re not quite developed enough to always be making those best choices. (T3)

Parents/guardians and teachers acknowledged that parents/guardians could learn about healthy sleep habits through school–home collaboration (e.g. emails, phone calls, parent–teacher interviews and students as change makers). Teachers encouraged parent/guardian involvement in school-based sleep promotion as they recognised that schools also educate parents/families.

With regards to administration and parent/guardian partnership, school administrative staff described how they had formal (phone calls) and informal conversations with parents/guardians about students’ sleep habits and some administrators shared resources on sleep with families. Teachers also checked in with students and families when students were struggling. As one administrator shared:

I think my role as a principal . . . is figuring out when kids aren’t regulated, and they are having difficulty in school . . . It’s my job to help them figure that out. And then that’s conversations with parents, and we have those conversations often about sleep. When are kids going to bed? How much sleep are they getting? Is there a routine in place? . . . I’d say almost all my teachers have had those conversations in their class with their kids. Pretty informally, but yes. (T17)

## Discussion

This secondary analysis brought together student, parent/guardian and teacher beliefs, behaviours, practices, strategies and recommendations to inform and strengthen school-based sleep promotion. Subthemes were identified within each CSH component: social and physical environment; teaching and learning; policy and partnerships and services. Students, parents and teachers had varying levels of insight on each CSH component within the secondary data sets. Teachers appeared to have the most insight followed by parents and students. Findings from the partner interviews can be used to inform and strengthen school-based sleep promotion using a CSH approach. CSH is most successful in shifting a school culture when all four components work collaboratively together (JCSH, 2016). Therefore, findings would best inform and strengthen school-based sleep promotion when implemented collectively across all four components of CSH.

A key finding from this study was that schools could foster a sleep promoting social and physical school environment by creating a culture of healthy sleep habits and by students influencing each other. According to students, parents/guardians, and teachers, schools could achieve this goal through role modelling, informal conversations, class discussions, and monthly campaigns. In a study examining teachers’ perceptions of the implementation of a CSH project, teachers perceived that part of their role as a teacher was role modelling (Storey et al., 2011). Teachers in a school-based health intervention study by Cargo et al. (2006) described role modelling as ‘setting a positive example for children and in doing so reinforcing intervention messages’ (p. 91).

To shift a school culture towards one that is health promoting, two studies (Neely et al., 2020; Storey et al., 2016) identified the essential conditions for taking a CSH approach. Within this secondary analysis, we found important overlaps between these essential conditions and our results. Notable areas of commonality included the need for ‘community support’ (i.e. collaboration between schools, teachers and parents/guardian to support healthy sleep habits; community partnerships; school–home collaboration is a critical partnership for healthy sleep); and ‘students as change makers’ (i.e. students influence each other). In our study, students were seen to have an important role in influencing the sleep behaviours of peers and family members, a finding that is well aligned with other research identifying students as change makers in promoting health behaviours (Bird et al., 2021; McKernan et al., 2019). Therefore, students as change makers and community support (i.e. school-home collaboration) are key components in the successful implementation of school-based sleep promotion when taking a CSH approach.

The social and physical environment of the school influences student’s health and well-being often through ‘hidden’ or ‘informal’ curricula within a school’s culture. This informal curricula is shaped by the school’s values (Langford et al., 2015) and can be used as a way to complement existing curricula requirements. Rather than only educating students about sleep through ‘hidden’ or ‘informal’ curricula, parents/guardians and teachers were supportive of the formal integration of sleep into the provincial school curricula despite an already overloaded curriculum. Currently, sleep is not an organising idea or learning outcome within the provincial curricula at the primary study sites; therefore, sleep is something that teachers can cover (or ignore) if they choose (LearnAlberta, 2022). If sleep were to be more formally integrated into the provincial curricula, it would help demonstrate the value of sleep and how adequate sleep benefits students’ social, physical and mental well-being. Integration of formal sleep education in Canadian schools has been recommended by other sleep researchers, as a means to improve student learning, well-being and health (Gruber et al., 2019).

In this study, teachers and parents/guardians advocated for collaboration between schools, teachers and parents/guardians. Students shared how they learned about sleep from school and home. Parents/guardians and teachers both identified school-home collaboration as a critical partnership to achieve healthy sleep habits for students. Therefore, in alignment with research by Epstein (Epstein, 2018; Epstein and Salinas, 2004), collaboration between teachers and parents/guardians would support student engagement in learning, and foster partnerships and services to support the promotion of healthy sleep behaviours. Establishing home, school and community partnerships has been identified as a priority for school communities (Epstein, 2018; Epstein and Salinas, 2004); however, the means to enable such partnership formation are less clear. Partners in this secondary analysis recommended good communication between teachers and parents/guardians when students were having sleep problems, schools teaching healthy sleep behaviours to support parents’/guardians’ efforts at home, schools sharing resources and contacts with parents/guardians to support healthy sleep habits, school-wide sleep awareness events and parent/guardian education on sleep. Research shows that strong collaborative relationships between schools and families benefit everyone (Epstein, 2018; Jones, 2020; Miller et al., 2018) and that parent/guardian knowledge of sleep and monitoring of children’s bedtime increased students’ likelihood of meeting sleep recommendations (Bird et al., 2021, 2023; McDowall et al., 2016). In addition, school-based sleep interventions have greater success when they involve collaborating with school partners (Gruber et al., 2016; Rey et al., 2020). Findings from Storey et al. (2016) have highlighted how ‘. . . in order to truly shift a school culture, all members of the school community need to play a role’ (p.5).

### Strengths and limitations

Strengths of this study included having two researchers analyse the data, the relatively large sample size, and range of partner perspectives. Our sample broadens the scope of potential impact of this research for those involved in the implementation of school-based sleep promotion. Given this study involved a secondary analysis, the interviewers did not specifically ask participants about the kind of partnerships that support students’ healthy sleep habits or about policies that help promote healthy sleep behaviours (i.e. delayed school start times, bus schedules). Therefore, limitations of this research were that it does not offer an accurate description of the community partners available to support school-based sleep promotion and lacks engagement with higher-level policy discussion. Interviews with school district leaders and school superintendents may yield different insights related to community partners (Bird et al., 2023) and school and district policies about sleep.

## Conclusion

Findings from the research lead us to suggest a number of strategies that can be used to shift school and community culture so as to promote healthy sleep habits. They include (1) fostering school-home collaboration through parent/guardian education and supporting students as change makers to influence each other and family members sleep behaviours; (2) formally integrating sleep**-**specific learning into relevant parts of the school curricula; (3) developing classroom, school and school district sleep-positive policy; (4) fostering collaborative teaching and learning about healthy sleep behaviours at school and home and (5) finding, fostering, developing community partnerships that can support healthy sleep habits in school communities.

Taken together, findings from this study provide direction to inform and strengthen school-based sleep promotion. The results provide insight into specific beliefs, behaviours, practices and recommendations for school communities. These tangible, action-oriented results build on the strengths that already exist in the school communities. By building on existing strengths in school communities, school-based sleep promotion taking a CSH approach has the potential to improve students’ and their families’ sleep and behaviours. When taken up and used appropriately, these findings have broad applicability to support comprehensive school-based sleep promotion efforts within Canada and globally.

## Supplemental Material

sj-docx-1-hej-10.1177_00178969241286660 – Supplemental material for Students’, parents’ and teachers’ perspectives on comprehensive school-based sleep promotion Supplemental material, sj-docx-1-hej-10.1177_00178969241286660 for Students’, parents’ and teachers’ perspectives on comprehensive school-based sleep promotion by Pamela Mellon, Genevieve Montemurro, Samuel (Sukmin) Yang, Lauren Sulz, Brian Torrance and Kate Storey in Health Education Journal
