# Supplementary material for: Students’, parents’ and teachers’ perspectives on comprehensive school-based sleep promotion
Source: Health Educ J. 2024 Sep 30;83(7):796–808. doi: 10.1177/00178969241286660 (PMC11530339; doi:10.1177/00178969241286660)
Supplement: sj-docx-1-hej-10.1177_00178969241286660 – Supplemental material for Students’, parents’ and teachers’ perspectives on comprehensive school-based sleep promotion [file sj-docx-1-hej-10.1177_00178969241286660.docx]

**Supplemental Table 1.** Comprehensive School Health (CSH) Component Definitions and Examples

| **CSH Component** | **Component Definition & Examples** |
| --- | --- |
| Social and Physical Environment | The social environment is the quality of the relationships among and between staff and students (Ever Active Schools, n.d.).  The social environment includes (JCSH, 2016):   - The quality of the relationships among and between staff and students in the school - The emotional well-being of students - Influenced by relationships with families and the wider community - Supportive of the school community in making healthy choices by building competence, autonomy, and connectedness   Social environments shape how students feel at school. They include (Alberta Health Services, n.d.):   - Positive relationships between students, their peers, and school staff (including virtual relationships) - Ways of welcoming and including all students within a classroom or learning cohort, and promoting belonging - Opportunities for students to be full and equal partners in decision-making about their school experience   The physical environment is the buildings, grounds, play space and equipment both in and surrounding the school. The physical environment should be safe, accessible and supportive of healthy choices (Ever Active Schools, n.d.).  The physical environment is (JCSH, 2016):   - The buildings, grounds, play space, and equipment in and surrounding the school - Basic amenities such as sanitation, air cleanliness, and healthy foods - Spaces designed to promote student safety and connectedness and minimize injury - Safe, accessible, and supportive of healthy choices for all members of the school community   Physical environments are spaces for learning, movement, and play. They include (Alberta Health Services, n.d.):   - Indoor spaces like classrooms, hallways, gymnasiums, cafeterias, and learning commons - Outdoor spaces like playgrounds, fields, outdoor classrooms, gardens, and pathways - Equipment and materials like furniture, kitchen appliances, lighting, and ventilation |
| Teaching and Learning | Teaching and learning encompasses formal and informal curriculum, resources and associated activities. It refers to the knowledge, understanding and skills needed for school communities to improve their health and enhance learning outcomes. Professional development opportunities for staff related to health and well-being also fall under this component (Ever Active Schools, n.d.).  Teaching and learning includes (JCSH, 2016):   - Formal and informal provincial/territorial curriculum, resources, and associated activities - Knowledge, understanding, and skills for students to improve their health and wellbeing and enhance their learning outcomes - Professional development opportunities for staff related to health and well-being   Teaching and learning involve the ways that both students and staff learn and practice health at school. It includes (Alberta Health Services, n.d.):   - Curriculum-based instruction and activities to develop, strengthen, and practice skills (including both in-person and virtual teaching) - Informal instruction that happens outside of traditional learning spaces, like through online social experiences, hallway or recess conversations, or extracurricular activities - Training for educators, including virtual and in-person workshops, presentations, webinars, mentorships, and collaboratives |
| Policy | Policy means the policies, guidelines and practices that promote and support student well-being and achievement, and shape a respectful, welcoming and caring school environment for all members of the school community (Ever Active Schools, n.d.).  Policies, guidelines, and practices that promote and support student well-being and achievement and shape a respectful, welcoming, and caring school environment for all members of the school community (JCSH, 2016).  Policies are written directives that influence school life. They include (Alberta Health Services, n.d.):   - Policies and standards set by Alberta Education, like the Daily Physical Activity Policy, Human Sexuality Education Policy, and Ministerial Order on Student learning - Board policies, administrative procedures, and regulations set by school authorities - Multi-year education plans or frameworks developed by school authorities - Annual development plans in schools - Student codes of conduct |
| Partnerships and Services | In a CSH context, partnerships and services are supportive working relationships with other community organizations. It includes health, education and other sectors working together to advance health; as well as community and school-based services that support health and wellness for all (Ever Active Schools, n.d.).  Partnerships are (JCSH, 2016):   - The connections between the school and students’ families - Supportive working relationships among schools, and among schools and other community organisations and representative groups - Health, education, and other sectors working together to advance school health   Partnerships are connections and relationships between schools and communities (Alberta Health Services, n.d.):   - Parents, caregivers, and other family members - Community organisations and groups - Elders and other community champions - Local facilities like recreation centres, libraries, and parks |
|  | Services are (JCSH, 2016):   - Community and school-based services that support and promote student and staff health and well-being. Services are: - Community and school-based services that support and promote student and staff health and well-being.   Services are supports offered by health professionals or social service providers in collaboration with schools, like (Alberta Health Services, n.d.):   - Immunisation programs for students - Public health inspections - Teacher training in school health promotion   School health information, resources and coaching |

Source definitions and examples are derived from the following established Comprehensive School Health framework guidelines:

Alberta Health Services. (n.d.). *The CSH Framework*. Retrieved January 18 from <https://schools.healthiertogether.ca/en/learn/the-csh-framework/>

Ever Active Schools. (n.d.). *Comprehensive School Health*. Retrieved January 18 from <https://everactive.org/comprehensive-school-health/>

JCSH. (2016). *What is Comprehensive School Health?* Pan-Canadian Joint Consortium for School Health

<https://www.jcsh-cces.ca/en/concepts/comprehensive-school-health/>
